# Early life and infant mental health: Reshaping assumptions in a southern field

**DOI:** 10.4102/jcmsa.v2i1.74

**Published:** 2024-05-31

**Authors:** Fiona Ross, Michelle Pentecost, Anusha Lachman

**Affiliations:** 1 Department of Anthropology, Faculty of Humanities, University of Cape Town, Cape Town, South Africa; 2 Department of Global Health and Social Medicine, Faculty of Social Health and Policy, Kings College, London, United Kingdom; 3 Department of Psychiatry, Faculty of Medical and Health Sciences, Stellenbosch University, Cape Town, South Africa

**Keywords:** early life, mental health, infants, global health, normal development, structural violence

## Abstract

Mental health is a priority area for global health, with a particular focus on well-being in majority of the world countries. Attention to early life demonstrates the significance of infant well-being for long-term health. International organisations such as the United Nations International Children’s Emergency Fund (UNICEF), World Health Organization (WHO), and the World Bank guidelines shape interventions in the majority world. At the same time, there are severe shortages of trained mental health personnel on the African continent and growing concerns about the potentially skewed evidence base that informs the science of interventions. Scholars across a range of disciplines are calling for attention to more diverse evidence sources; for better understandings of the syndemic interactions that shape mental health and for interventions that take account of local ideals while retaining a strong evidence base. As questions of how best to secure infant well-being and the adequacy of knowledge surrounding it emerges with growing force on the global scene, it is critical that the full range of infants’ worlds are represented in scholarship. What do exposures to structural violence, interpersonal violence, social assault, and environmental insult mean for our understanding of ‘normal’ development both in our context and globally? What are the dangers of not accounting for these exposures? What evidence bases matter? How do we know? These are critical questions. They arise in the context of limited, under-resourced and often poorly supported opportunities for adequate screening, early recognition, and suitable interventions for both infants and caregivers in Africa.

The World Health Organization (WHO) prioritises mental health and well-being as intrinsic to a person’s overall ability to cope with life stressors, function effectively in a work and social environment, and to optimally contribute within their communities. This emphasises the importance of making mental health a priority area for global health especially in majority of the world settings.^1,2^ Work within the first 1000 days focussing on early life adversity and resilience demonstrates the significance of infant well-being for long-term health. International organisations such as the United Nations International Children’s Emergency Fund (UNICEF), WHO and the World Bank are developing guidelines that shape interventions in the majority world – a framing we prefer to ‘Global South’ as it includes those in the north who have been dispossessed through systemic racism and other forms of structural violence.^3^ At the same time, there are severe shortages of trained mental health personnel on the African continent and growing concerns about the potentially skewed evidence base that informs the science of interventions.

Scholars across a range of disciplines^2,4^ are calling for attention to more diverse evidence sources for better understandings of the syndemic interactions that shape mental health and for interventions that take account of local ideals while retaining a strong evidence base.

As questions of how best to secure infant well-being and the adequacy of knowledge surrounding it emerge with growing force on the global scene, it is critical that the full range of infants’ worlds are represented in scholarship. What presuppositions about ‘the human’, personhood and relationship underpin our models? What do exposures to structural violence, interpersonal violence, social assault, and environmental insult mean for our understanding of ‘normal’ development both in our context and globally? What are the dangers of not accounting for these exposures? What evidence bases matter? How do we know?

These are critical questions. They arise in the context of limited, under-resourced and often poorly supported opportunities for adequate screening, early recognition, and suitable interventions for both infants and caregivers in Africa.

UNICEF’s projection that Africa’s child population will increase to 1 billion by the year 2030, highlights the urgency need to ensure universal access to healthcare and to train personnel to meet service demands. An interrogation of the available educational offerings, the applicability or generalisability of existing screening measures and that the skillset of the professionals need to be reviewed if we are to innovatively approach an overwhelming need within a severely constrained health and allied care system.

Innovation requires creativity. Effective intervention requires a wider evidence base and critical assessment of scholarship’s underlying premises to ensure that experiences are justly represented and that the evidence base on which interventions are made are adequate and responsive to majority population. Along with other majority world scholars,^5,6^ we are concerned about the publication practices that underpin the production of scientific data and interventions. There are two key questions: what is published and how?

Recent critiques of what has come to be known as western, educated, industrialised, rich, and democratic (WEIRD) psychology have demonstrated the culturalist and materialist biases in knowledge formations imagined to be universal. The same knowledge formations that inform many contemporary interventions in infant and family life. This does not mean that the findings of such studies cannot be generalised. It points rather to a need to examine the underpinning premises of knowledge-making rather than presume its validity. It also suggests that we need to check our assumptions and diversify our understanding of how context and development interact. Do we accept that ‘the infant’ is universally ‘the same’, generic and pre-cultural? Do we ask whether it makes a difference that a child is born in a highly industrialised, highly stratified society or one characterised by egalitarian relations? Does cultural difference matter? If so, how? That is, should we account for difference, and if so, how do we do so without essentialism? Scholarly work predicated on Euro-American norms may not adequately represent the experiences of infants in lower- and middle-income countries, where family structures, social support systems, economic and political conditions, and ideas about relatedness may differ from the narrow specifics of WEIRD societies and where mental health is particularly at risk because of histories of intergenerational trauma, extraction and dispossession. This difference is usually expressed in terms of disadvantage, as observed by Scheidecker et al.,^7^ and Lachman et al.^8^ It is also true, although unacknowledged, that such societies offer potentials that are as yet unrecognised, including models of relationality that could greatly enrich our understanding of what it is to be human at this point in time.

Equally important, current publication practices exclude this knowledge from the evidence base that shapes policy. Editorial practices matter. Several key journals in the field of infant mental health have minimal or even no representation by members of the majority world even though research published in these journals informs the evidence base on which policy for the majority world is premised. While the presence of majority world scholars is not a guarantee of either diversity in research or of a more robust scholarship, the absence of representation repeats exclusionary practices and hierarchies of knowledge production, and runs the risk of precluding generative critique. Critique does not mean that the existing scholarship is intrinsically misguided.^4^ Rather, it is necessary to ensure that normative assumptions in our disciplines do not diminish our humanity. The idea is to build a fuller picture.

## On not throwing the baby out with the bathwater

Evidence-based research matters. At the same time, contexts and histories differ, with direct implications for how well-being is understood, distributed, experienced, and supported.^9^ While there is a wealth of knowledge about cross-cultural validation, more work, of a different kind, is necessary. Values, stated or implicit, underpin our models. What are they and how well do they work for our context and the futures we envisage? We have a robust science of infancy and early development that now needs careful and critical evaluation *in situ*. This is more than just ‘cross-cultural translation and validation’, important as they are. Rather, we are suggesting careful attention to which approaches and tools work most effectively in identifying problems and supporting well-being. Scholars of mixed race, indigenous peoples, and activists have long worked on these dimensions and there are powerful examples from which inspiration can be drawn. Similarly, careful assessment and comparison of existing tools^10^ can generate important insights about what works best in specific contexts and can also highlight gaps in knowledge, foreground erroneous presumptions (including about the nature of the infant, the context, and the relationship) and enable work that is considerate of history’s effects, contextual features, and universal features of infancy and care.^11^

As we navigate an exciting and evolving field of what early life and infant mental health and well-being means and how it is represented on the African continent, we encourage the embracing of local scholarly expertise and the addressing of exclusionary scholarly practices. We propose that scientific publications and discourses ensure that scholarship is:

- Anti-racist, anti-sexist, and anti-colonial in its praxis, including in publication.
- Inclusive at a range of levels, including in terms of an understanding of the diversity of human experiences and values; non-exploitative scholarly collaborations and a wider range of disciplinary knowledge contributions. Diverse citational practices^12^ and the composition of editorial and advisory boards also promote inclusivity.
- Consideration needs to be given to the limits of knowledge and the histories of exclusion that have shaped disciplinary knowledge. It requires an awareness of potential power relations, including in research encounters and the effects of knowledge practices as they enter the world through policy and practice.

Africa’s population is the youngest in the world. It makes sense to ensure that research and interventions support the forms of life and relationality that have value here. In the face of polycrises – growing global inequality, climate change, and massive demographic shifts, it is imperative that we draw on as wide a range of resources as possible to secure well-being. Diversifying the knowledge base on which interventions are built, ensuring that the experiences of the majority world are taken into account, and promoting inclusive publication practices are the first steps towards a more just and sustainable future.

## References

[1] Becker AE, Raviola G, Kleinman A. Introduction: How mental health matters. Daedalus. 2023;152(4):8–23. 10.1162/daed_e_02029

[2] Sodi T, Abas M, Abdulaziz M, et al. A research agenda for mental health in sub-Saharan Africa. Nat Med. 2024;30:616–617. 10.1038/s41591-023-02779-6 38355971

[3] Khan T, Abimbola S, Kyobutungi C, Pai M. How we classify countries and people – And why it matters. BMJ Glob Health. 2022;7:e009704. 10.1136/bmjgh-2022-009704 PMC9185389 35672117

[4] Oppong S. Epistemological allyship. Psychol Dev Soc J. 2023;35(1):69–86. 10.1177/09713336231152301

[5] Draper CE, Barnett LM, Cook CJ, et al. Publishing child development research from around the world: An unfair playing field resulting in most of the world’s child population under-represented in research. Infant Child Dev. 2023;32:e2375. 10.1002/icd.2375

[6] Naidu T. Says who? Northern ventriloquism, or epistemic disobedience in global health scholarship. Lancet Glob Health. 2021;9:e1332–e1335. 10.1016/S2214-109X(21)00198-4 34416216

[7] Scheidecker G, Chaudhary N, Keller H, Mezzenzana F, Lancy DF. ‘Poor brain development’ in the global south? Challenging the science of early childhood interventions. Ethos. 2023;51:3–26. 10.1111/etho.12379

[8] Lachman A, Berg A, Ross F, Pentecost M. Infant mental health in southern Africa: Nurturing a field. Lancet. 2021;398(10303):835–836. 10.1016/S0140-6736(21)00998-3 34058135

[9] Behague D, Tawiah C, Rosato M, Some T, Morrison J. Evidence-based policy-making: The implications of globally-applicable research for context-specific problem-solving in developing countries. Soc Sci Med. 2009;69(10):1539–1546. 10.1016/j.socscimed.2009.08.006 19781839

[10] Dawson N, Bain K, Mesman J. Observing maternal sensitivity in a South African township: An exploratory study into behavioral features using different measures. Attach Hum Dev. 2021;23(2):150–163. 10.1080/14616734.2020.1828531 33016856

[11] Quinn N. Universals of child rearing. Anthropol Theory. 2005;5(4):477–516. 10.1177/1463499605059233

[12] Makhulu A-M. Citing Black Women: From citational refusal to recognition. Cult Anthropol. 2022;37(2):214–224. 10.14506/ca37.2.06

